# Supplemental effects of mixed ingredients and rice bran on the growth performance, survival and yield of Nile tilapia, *Oreochromis niloticus* reared in fertilized earthen ponds

**DOI:** 10.1186/s40064-015-1643-x

**Published:** 2016-01-04

**Authors:** S. M. Limbu, A. P. Shoko, H. A. Lamtane, M. A. Kishe-Machumu, M. C. Joram, A. S. Mbonde, H. F. Mgana, Y. D. Mgaya

**Affiliations:** Department of Aquatic Sciences and Fisheries, University of Dar es Salaam, P. O. Box 35064, Dar es Salaam, Tanzania; Department of Biology, School of Life Sciences, East China Normal University, Shanghai, China; Tanzania Fisheries Research Institute, Headquarters, Dar es Salaam, Tanzania; Department of Animal Science and Production, Sokoine University of Agriculture, Morogoro, Tanzania; Kilombero District Council, Ifakara, Morogoro, Tanzania; Tanzania Fisheries Research Institute, Kigoma Centre, Kigoma, Tanzania

**Keywords:** Mixed feed ingredients, Rice bran, Semi-intensive culture, *Oreochromis niloticus*, Phytoplankton

## Abstract

Unaffordability of commercial feeds to semi-intensive Nile tilapia, *Oreochromis niloticus* farmers has led to reliance on supplemental feeding and fertilization for nutrition of their fish without a scientific basis. This study compared the growth, survival, condition factor and yield performance of *O.niloticus* fed on mixed ingredients (MI) and rice bran alone (RB) diets reared in fertilized earthen ponds in small scale farmers’ ponds. The study also determined phytoplankton composition, biomass and abundance in the experimental ponds. Quadruplicate ponds were stocked with *O. niloticus* and African sharptooth catfish, *Clarias gariepinus* at a stocking ratio of 1:3. The initial mean weights of *O. niloticus* and *C. gariepinus* were 14.62 ± 0.61 and 20.34 ± 1.44 g respectively. The fish were fed on MI and RB diets with protein content of 184.74 and 126.06 g kg^−1^ respectively for 270 days. The results showed that growth performance, survival rate and condition factor of *O.niloticus* were not affected by either feeding with MI or RB diet (p > 0.05). Moreover, there were no significant differences obtained in phytoplankton biomass and abundance in *O.niloticus* ponds fed on the two diets (p > 0.05). Furthermore, feeding *O.niloticus* either on MI or RB diet did not affect the net and gross fish yields (p > 0.05). The study revealed that the use of either MI or RB diet does not significantly affect the performance of *O.niloticus* cultured in semi-intensive earthen ponds where natural food organisms are an integral part of the culture system. Rice bran could be used for semi-intensive culture of *O.niloticus* in fertilized ponds to boost the production based on its availability and low cost.

## Background

The majority of fish farmers in developing countries practise semi-intensive culture of Nile tilapia (*Oreochromis niloticus* L.) by feeding them with on-farm made feeds due to unavailability and high cost of commercial feeds. In Tanzania, over 95 % of fish farmers practise semi-intensive mixed-sex culture of *O. niloticus* in earthen ponds (Kaliba et al. 2006). To a small extent, farmers stock *O.niloticus* and African sharptooth catfish (*Clarias gariepinus*) in polyculture system (Shoko et al. 2014a, b; Limbu et al. 2015). In both cases, the culture of *O.niloticus* in earthen ponds entirely depends on supplementary feeds such as rice, maize and wheat bran; and sunflower and cotton seed cakes with or without fertilization using animal manure. The latter is commonly used where pond fertilization is practised.

Previous studies have shown that feeding is one of the main factors required for faster growth and higher yield of cultured *O.niloticus* (Abdel-Tawwab et al. 2007, 2010; Limbu and Jumanne 2014). When cultured in semi-intensive earthen ponds, *O.niloticus* has been shown to thrive on supplementary diets made from agro-byproducts augmented with fertilization based on its omnivorous feeding behaviour (Charo-Karisa et al. 2006; Abdel-Tawwab et al. 2007). Different plant based diets such as wheat bran (Liti et al. 2005), maize, wheat and rice brans (Liti et al. 2006), cotton and sunflower seed cakes (Aanyu et al. 2014) and sunflower oil (Carbonera et al. 2014) have all been studied with varying results on their effects on growth, survival and yield on *O. niloticus*. To promote faster growth and higher yields, ponds in which *O. niloticus* is fed on plant-based diets are occasionally fertilized using different types of organic manures to stimulate growth of phytoplankton (Rukera et al. 2012). Phytoplankton availability has a synergistic effect in promoting high growth performance, survival rate and yield of *O.niloticus* cultured in semi-intensive ponds (Li and Yakupitiyage 2003).

Feeding *O. niloticus* in semi-intensive ponds varies greatly in different countries depending on socio-economic status and knowledge of farmers (Yakubu et al. 2012). *O. niloticus* farmers in Tanzania commonly use simple mixture or single ingredient for feeding their fish (Kaliba et al. 2006) without any proven scientific guide. Thus, there is a need to provide viable and low-cost scientific based feeding information to *O.niloticus* farmers for improved production using resources available in their local settings.

This study assessed the growth performance, condition factor, survival and yield of *O.niloticus* fed on MI and RB diets. The study also compared phytoplankton composition, biomass and abundance in ponds fed with MI and RB diets. It was hypothesized that, growth performance, condition factor, survival and yield of fish as well as phytoplankton abundance and biomass will be significantly higher for MI compared to RB treatments.

## Methods

### Study site

The study was conducted at Lufulu village in Kilombero District about 110 km from Ifakara town located at 8°31′0″ South, 35°56′0″ East in Morogoro region, Tanzania. The site is characterized by a rainy season from November to May and a dry season from June to October (Balama et al. 2013). The site receives an annual rainfall of between 1200 and 1800 mm and the temperature ranges from 25 to 32 °C (Sumaye et al. 2013). The main economic activities at the study site are agriculture include crop framing (rice, maize and sugar) complemented with fish farming.

### Experimental design

A participatory on-farm study was designed to assess growth performance, condition factor, survival, yield of *O.niloticus* fed on MI and RB diets and phytoplankton species composition, biomass and abundance. Eight fish ponds owned and operated by four independent fish farmers within Lufulu village were used in the present study. Quadruplicate ponds were stocked with *O.niloticus* and African sharptooth catfish, *Clarias gariepinus* at a stocking ratio of 1:3. The initial mean weights (±standard error, SE) of *O.niloticus* and *C.gariepinus* were 14.62 ± 0.61 and 20.34 ± 1.44 g respectively. *Clariasgariepinus* was stocked intentionally to control the population size of *O.niloticus* recruits resulting from prolific reproduction (Limbu et al. 2015). The two fish species were stocked at a density of three individuals per square metre and reared for 270 days. One quadruplicate group of the two fish species were fed on MI diet whereas the other group was fed on RB diet. The MI diet was made using locally available sunflower seed cake and rice bran mixed at 760.00 and 240.00 g kg^−1^ of dry feed respectively. Rice bran (RB) is the vitamin-rich outer layer that surrounds the endosperm of whole grain brown rice. Although rice bran comprises about 8 % of total weight of rice kernel, it accounts for 60 % of the nutrients (Saunders 1985). The formulation and proximate composition of the two diets used are shown in Table 1.

**Table 1:** Formulation and proximate composition (dry matter basis) of diets used in the study

Ingredients	Diets formulation (g kg^−1^)	
	Mixed ingredient (MI) diet	Rice bran alone (RB) diet
Sunflower seed cake	760.00	0.00
Rice bran	240.00	1000.00
Total	1000.00	1000.00
Proximate composition of diets		
Dry matter	917.02	916.70
Crude protein	184.74	126.06
Crude fibre	188.09	162.15
Crude fat	102.95	129.10
Ash	67.28	59.34
Carbohydrate	373.96	440.05
Gross energy (kcal g^−1^)^a^	17.31	19.95

^a^Calculated using the factors: carbohydrates, 4.1 kcal g^−1^, protein, 5.4 kcal g^−1^ and lipids, 9.5 kcal g^−1^ (Tacon 1990)

The sunflower seed cake for making the MI diet was purchased from local suppliers at Ifakara. Rice bran was obtained from nearby rice millers free of charge. The two ingredients were separately ground using a hammer mill with a screen size of 0.8 mm, weighed and mixed in the required proportions to form a mash. The RB diet was prepared using the rice bran mash obtained during the preparation of MI diet above. The resulting mashes were applied at a daily ration of 5 % average body weight of *O.niloticus* at the start of the study. In order to reflect decrease in metabolic rate as fish grow (Sousa et al. 2012), the feeding rate was reduced to 4 and 3 % when the average weight of *O.niloticus* reached >50 and >100 g respectively. Fish were fed by hands twice daily between 0900 and 1000 h and 1500 and 1600 h by broadcasting at the periphery of each pond. To ascertain proper feeding regimes, sampling frequencies and minimize variations caused by daily management regimes in different fish farmers’ ponds, the authors collaborated closely with local fish farmers. Proximate analyses of the diets and cattle manure used were conducted according to the Official Methods of Analysis of the Association of Official Analytical Chemists (AOAC 1990). Energy was estimated based on amount released per gram of 4.1 kcal g^−1^ for carbohydrates 5.4 kcal g^−1^ for protein and 9.5 kcal g^−1^ for lipids (Tacon 1990).

Apart from feeding, the fish ponds were fertilized by using cattle manure at a rate of 280 kg ha^−1^ week^−1^ (Garg and Bhatnagar 1999). The manure was applied by using a bamboo wooden crib constructed at the water inlet in each pond. The chemical composition of the dry cattle manure was moisture 755.00 g kg^−1^, organic matter 222.00 g kg^−1^, total nitrogen (N) 9.20 g kg^−1^, total phosphorus (P_2_O_5_) 4.30 g kg^−1^, potassium 4.00 g kg^−1^ and calcium 5.50 g kg^−1^.

### Data collection

Growth parameters were monitored by randomly sampling 30 individuals of *O. niloticus* on a monthly basis. The fish were individually weighed by using a sensitive weighing balance. Body length was measured to the nearest 0.01 cm using a measuring board and immediately returned to the water of the same fish pond. After 270 days of culture, ponds were drained and all *O. niloticus* and *C. gariepinus* were harvested, counted for percentage survival determination and batch weighed for determination of gross yield (GFY, kg ha^−1^) by using a kitchen weighing scale. A sample of 30 *O. niloticus* drawn randomly from each pond was weighed individually to determine the final mean body weight (FMW). The data obtained were used to calculate growth, condition factor, percentage survival and yield indices using the following formulae:

1. Mean weight gain, $({\text{MWG,}}\;{\text{g}})\; = \;{\text{Wf}}\; - \;{\text{Wi}}$

where Wf and Wi are previous sampling mean initial and the current measured mean weights respectively.

2. Daily weight gain, $({\text{DWG,}}\;{\text{g day}}^{ - 1} )\; = \;\frac{{{\text{Wf}}\; - \;{\text{Wi}}}}{{{\text{Time}}\; ( {\text{days)}}}}$
3. Specific growth rate, $\left( {{\text{SGR}}\;{\text{\%}}} \right)\; = \;\left( {\frac{{\ell {\text{nWf}}\; - \;\ell {\text{nWi}}}}{{{\text{Time}}\; ( {\text{days)}}}}} \right)\; \times \;100$
4. The condition factor, ${\text{K}}\; = \;\left( {\frac{\text{W}}{{{\text{L}}^{ 3} }}} \right)\; \times \;100$

where W and L are individual weight (g) and length (cm) of *O. niloticus* respectively.

5. Percentage survival $$ ({\text{SR,}}\;{\text{\%)}} = \left( {\frac{{{\text{Fh}}\;}}{\text{Fi}}} \right)\; \times \; 1 0 0 $$

where Fh and Fi refer to final fish harvested and initial stocked number of *O. niloticus* respectively.

6. Net fish yield $({\text{NFY, kg ha}}^{ - 1} )\; = \;\frac{{{\text{W}}_{\text{h}} \; \times \;{\text{W}}_{\text{s}} }}{\text{A}}$

where W_h_ = total weight of fish harvested (kg), W_s_ = total weight of fish stocked (kg), A = pond area (ha).

7. Gross annual yield $({\text{GAY, kg ha}}^{ - 1} {\text{ year}}^{ - 1} )\; = \;\frac{{{\text{GFY}}\; \times \; 3 6 5}}{\text{Time (days)}}$
8. Net annual yield $({\text{NAY, kg ha}}^{ - 1} {\text{year}}^{ - 1} )\; = \;\frac{{{\text{NFY}}\; \times \; 3 6 5}}{{{\text{Time}}\; ( {\text{days)}}}}$

### Water quality, phytoplankton species composition, biomass and abundance

Water temperature, pH and dissolved oxygen (DO) were measured twice per day between 0800 and 0900 h and from 1600 to 1700 h for a period of nine months by using a multi-probe kit (model KTO, HQ, 40D PHC 101-LD 101-01 by Hach Company Ltd, USA). Sampling for phytoplankton composition, abundance and biomass was conducted every month for 5 months. In each pond, 20 litres of water samples were taken using a van Dorn sampler and filtered through a phytoplankton net (30 μm mesh size) for quantitative and qualitative analyses of phytoplankton. From this sample, 20 ml aliquots were fixed with Lugol’s solution prior to laboratory analysis. The samples were preserved in 2.5 % formaldehyde and transported in a cool box to Tanzania Fisheries Research Institute (TAFIRI) laboratory at Kigoma Centre in Kigoma, Tanzania for analysis.

In the laboratory, identification and counting of phytoplankton species were done using an inverted microscope at 400× magnification according to morphological criteria using available freshwater phytoplankton keys given by Utermohl (1958) and John et al. (2002). All specimens were distributed over at least two transects from the sedimentation chamber and counted. Different phytoplankton groups were counted as numbers of filaments, colonies and cells depending on the nature of the group. Phytoplankton species identified in a given sample was used to estimate its composition. Phytoplankton biomass was estimated indirectly using chlorophyll *a* analysis (Vörös and Padisák 1991). Water samples were filtered through a 0.45 μm Millipore membrane filters and extracted in 90 % acetone overnight at 4 °C. Chlorophyll *a* concentration was measured using a Shimadzu 1201 Spectrophotometer according to Parsons et al. (1984). Phytoplankton abundance was calculated according to Greenberg et al. (1992) using the formula:

9. Abundance = $$\frac{{{\text{C}}\;\, \times \,\;{\text{At}}\;\, \times \;\;{\text{v}}}}{{{\text{Af}}\;\, \times \,\,{\text{F}}\,\; \times \;\;{\text{V}}\;\; \times \;\;{\text{Vi}}}}\;$$

where, C = number of organisms counted, At = total bottom area of settling chamber (mm^2^), v = volume of concentrated sample (20 ml), Af = area of field (mm^2^), F = number of fields counted, V = volume of sample observed (2 ml) and Vi = volume of the sedimented sample.

### Statistical analyses

Results are presented as mean ± standard error (SE) and data were tested for homogeneity of variance using Levene’s test to guard against violation of the assumptions of parametric statistics. After confirming homogeneity of variances, a two-sample *t* test was utilized to test for significant differences in growth performance, condition factor, percentage survival, yield and water quality parameters between *O.niloticus* fed on the MI and RB diets. Similarly, t-test was used to compare the phytoplankton biomass and abundance in *O.niloticus* ponds fed on the MI and RB diets. Percentage data were arcsine-transformed prior to t-test. Results with p ≤ 0.05 were considered statistically significant (Zar 2010). All statistical analyses were performed using SPSS for Windows version 20 (SPSS, Inc).

## Results

### Water quality parameters and phytoplankton composition, biomass (chlorophyll *a*) and abundance

**Fig. 1:**
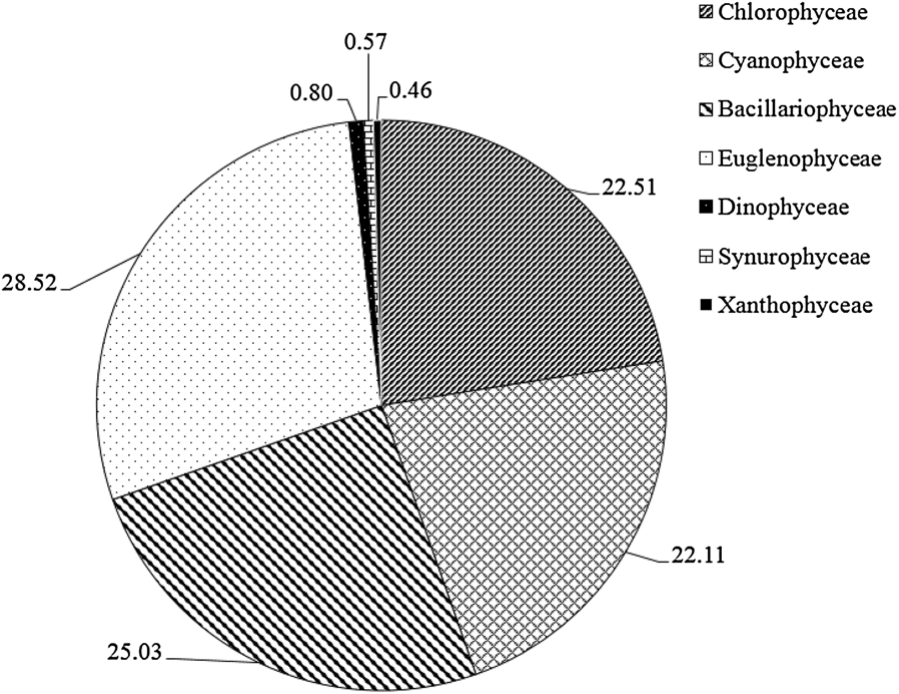
The percentage composition of phytoplankton groups recorded in *O.* *niloticus* ponds fed on the mixed ingredients (MI) and rice bran alone (RB) diets during the study period

Water temperature, dissolved oxygen and pH were found to be almost similar between treatments (Table 2). The water quality parameters did not differ significantly between the two diets during the study (p > 0.05). Results on phytoplankton composition recorded seven main groups dominated by Euglenophyceae (28.52 %) followed by Bacillariophyceae (25.03 %), Chlorophyceae (22.51 %) and Cyanophyceae (22.11 %). The percentage compositions of the other phytoplankton groups were less than one percent (Fig. 1). The evaluation of phytoplankton biomass (chlorophyll *a*) and total abundance revealed no significant differences between the two dietary treatments (t_(38)_ = 0.246, p = 0.807) and t_(152)_ = 0.884, p = 0.378) respectively.

**Table 2:** Water quality parameters, phytoplankton biomass (chlorophyll *a*) and total abundance in Nile tilapia, *Oreochromis* *niloticus* ponds under mixed ingredients (MI) and rice bran (RB) treatments during the study period

Parameter	Diet type	
	Mixed ingredients (MI) diet	Rice bran alone (RB) diet
Temperature (°C)	28.56 ± 0.68	28.19 ± 0.73
Dissolved oxygen (mg L^−1^)	5.16 ± 0.51	4.75 ± 0.70
pH	6.34 ± 0.07	6.20 ± 0.08
Chlorophyll *a* (µg L^−1^)	133.65 ± 39.22	121.29 ± 31.45
Total phytoplankton abundance (individuals L^−1^)	10438.13 ± 1513.40	8913.30 ± 873.22

All measured parameters were not significantly different between the dietary treatments (temperature, t_(54)_ = 0. 365, p = 0.716; dissolved oxygen, t_(54)_ = 0.480, p = 0.634; pH t_(54)_ = 1.252, p = 0.216); chlorophyll *a* (t_(38)_ = 0.246, p = 0.807) and phytoplankton abundance (t_(38)_ = t_(152)_ = 0.884, p = 0.378)

**Fig. 2:**
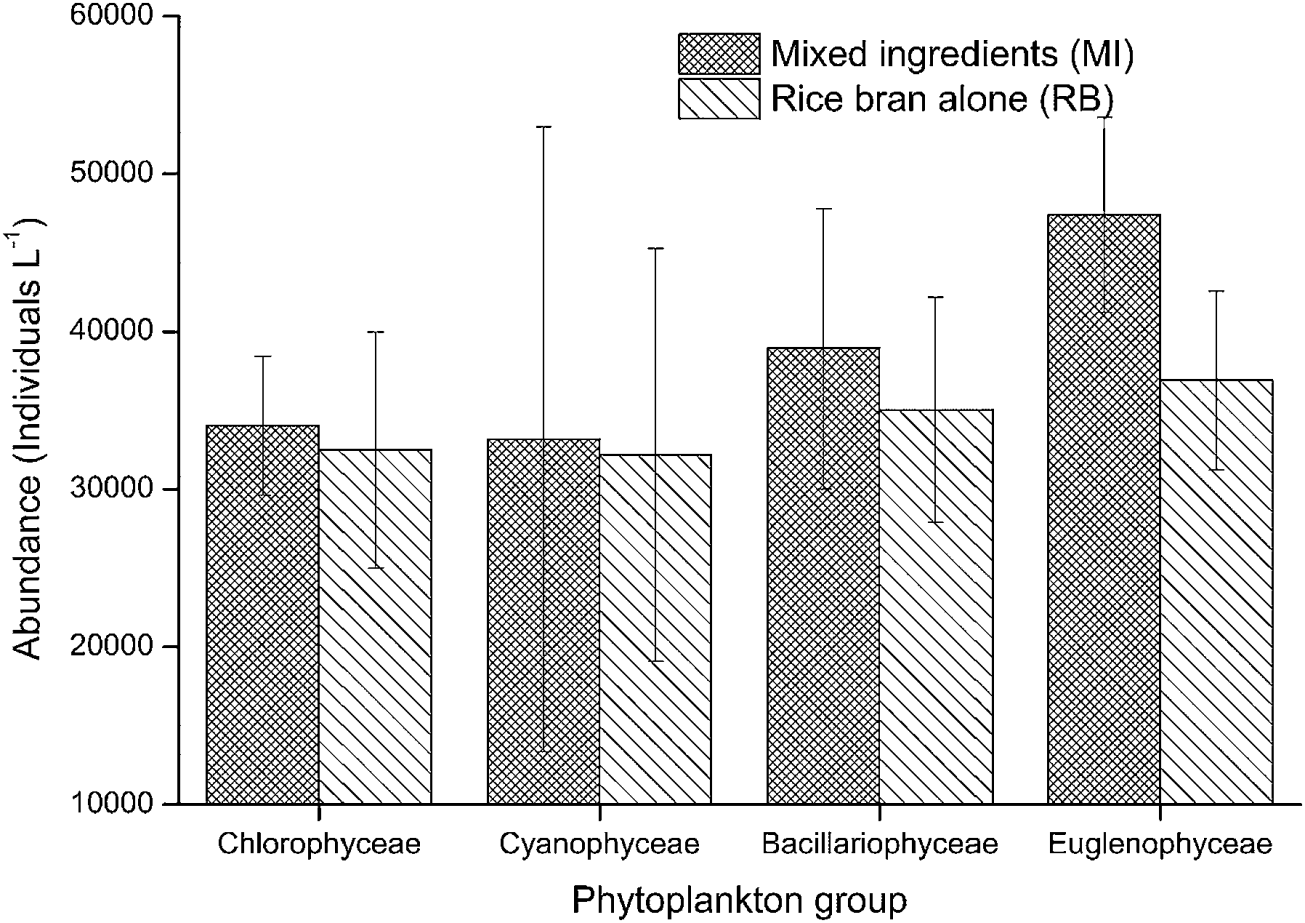
Abundance of main phytoplankton groups in Nile tilapia, *Oreochromis niloticus* ponds fed with mixed ingredients (MI) and rice bran alone (RB) diets. Abundance of each phytoplankton group was not significant different between *O. niloticus* fed on MI and RB diets (Chlorophyceae, t_(8)_ = 0.175, p = 0.865; Cyanophyceae, t_(8)_ = 0.043, p = 0.967; Bacillariophyceae, t_(8)_ = 0.341, p = 0.742; Euglenophyceae, t_(8)_ = 1.253, p = 0.246; and Dinophyceae t_(8)_ = 0.341, p = 0.742)

To understand which groups contributed more to total abundance, individual phytoplankton groups were analyzed. The abundance of Chlorophyceae was 34,022.65 ± 4417.19 and 32,499.24 ± 7477.50 individuals L^−1^ in ponds with *O.niloticus* fed on MI and RB diets respectively (Fig. 2). The abundance of Cyanophyceae was 33,176.31 ± 19,830.97 and 32,160.71 ± 13,084.01 individuals L^−1^ in ponds with *O.niloticus* fed with MI and RB diets respectively. Bacillariophyceae were 38,931.39 ± 8904.63 and 35,038.25 ± 7129.33 individuals L^−1^ in ponds with *O.niloticus* fed with MI and RB diets respectively. Feeding *O.niloticus* with MI and RB diets resulted in Euglenophyceae abundance of 47,394.73 ± 6178.82 and 36,900.18 ± 5655.90 individuals L^−1^ respectively. The abundance of phytoplankton groups identified was not significant different between the ponds under MI and RB diets (p > 0.05). However, relatively higher abundance was recorded in ponds with fish fed on MI diet compared to those fed on RB diet.

### Growth performance, condition factor and percentage survival

The final mean weight was 128.32 ± 7.32 g for *O.niloticus* fed on MI diet and 115.06 ± 6.53 g for those fed on RB diet. Feeding *O.niloticus* on either MI or RB diet did not affect significantly the final mean weights (t_(6)_ = 1.353, p = 0.225). The results on growth performance parameters revealed that mean weight gain, daily weight gain and specific growth rate were not significant different between *O*. *niloticus* fed on MI and RB diets (p > 0.05; Table 3). Similarly, condition factor of *O*. *niloticus* was also not statistically different in both treatments (t_(70)_ = −1.267, p = 0.209; Table 3). Furthermore, percentage survival of *O*. *niloticus* was not affected in any case by the diet type used in the present study (t_(6)_ = −1.267, p = 0.209; Table 3). However, with exception of condition factor, all other growth related parameters were comparatively higher for *O. niloticus* fed on MI than RB diet.

**Table 3:** Growth performance parameters of Nile tilapia *Oreochromis* *niloticus* fed on the mixed ingredients (MI) and rice bran (RB) diets during the study period

Growth parameter	Diet type	
	Mixed ingredients (MI) diet	Rice bran alone (RB) diet
Initial mean weight (g)	14.62 ± 0.61	14.62 ± 0.61
Final mean weight, FMW (g)	128.32 ± 7.32	115.06 ± 6.53
Mean weight gain, MWG (g)	12.63 ± 1.00	11.16 ± 0.88
Daily mean weight gain, DWG (g day^−1^)	0.43 ± 0.03	0.38 ± 0.03
Specific growth rate, SGR (%)	0.92 ± 0.15	0.89 ± 0.14
Condition factor (K)	1.85 ± 0.02	1.89 ± 0.02
Percentage survival (%)	82.60 ± 4.53	81.54 ± 6.18

All measured growth performance parameters were not statistically different (MWG, t_(70)_ = 1.112, p = 0.270; DWG, t_(70)_ = 0.939, p = 0.351 and SGR, t_(70)_ = 0.162, p = 0.872)

### The yields of *Oreochromisniloticus* and *Clarias gariepinus*

The yields for *O.niloticus* and *C. gariepnus* were comparatively higher for fish fed on MI than RB diet (Table 4). The results on yield parameters showed that, neither rearing *O.niloticus* on MI nor RB diet affected the net yields (t_(6)_ = −0.530, p = 0.615) and gross yields (t_(6)_ = −1.242, p = 0.261). Similarly, *C. gariepinus* net yields (t_(6)_ = 4.018, p = 0.658) and gross yields (t_(6)_ = 5.854, p = 0.538) were not affected by the two diets used.

**Table 4:** The yield parameters for Nile tilapia, *Oreochromis* *niloticus* and African sharptooth catfish, *Clarias gariepinus* fed on the mixed ingredients (MI) and rice bran (RB) diets during the study period

Fish species	Yield parameters	Diet type	
		Mixed ingredients (MI) diet	Rice bran alone (RB) diet
*Oreochromis niloticus*	Net fish yield (kg ha^−1^)	2559.16 ± 647.02	2201.50 ± 193.35
	Net annual yield (kg ha^−1^ year^−1^)	3459.61 ± 874.67	2976.10 ± 261.39
	Gross fish yield (kg ha^−1^)	3649.31 ± 910.04	2493.96 ± 193.35
	Gross annual yield (kg ha^−1^ year^−1^)	4933.32 ± 1230.24	3371.47 ± 261.39
*Clarias gariepinus*	Net fish yield (kg ha^−1^)	2693.57 ± 1345.78	1997.42 ± 562.84
	Net annual yield (kg ha^−1^ year^−1^)	3638.58 ± 1819.29	2700.22 ± 1521.75
	Gross fish yield (kg ha^−1^)	2745.83 ± 528.64	2212.68 ± 620.04
	Gross annual yield (kg ha^−1^ year^−1^)	3711.96 ± 714.64	2991.22 ± 838.20

All measured yield parameters were not statistically different (p > 0.05)

## Discussion

The water quality parameters obtained in the current study were not affected by the types of diets used. None of the parameters measured differed significantly between the ponds in which *O.niloticus* were fed on MI and RB diets. The water quality parameters obtained are comparable with those reported by Abdel-Tawwab et al. (2007), Rukera et al. (2012) and Oo et al. (2014) for *O.niloticus* reared in earthen ponds. The water quality parameters measured in the present study were within the optimum ranges required for survival and growth of *O.niloticus*. The main water quality parameters for optimal survival and growth of *O.niloticus* are temperature ranging from 25.0 to 30.0 °C, dissolved oxygen 4.0–8.0 mg/L and pH 6.5–9.0 (Suresh 2003; Hussain 2004; Shahabuddin et al. 2012). Although pH was slightly low (6.34 ± 0.07 to 6.20 ± 0.08) in pond water with MI and RB respectively, the levels recorded are not worrisome because *O.niloticus* can successfully survive, grow and tolerate pH range of between 4.00 and 11.00 (El-Sayed 2006). Nevertheless, farmers are advised to maintain water quality parameters including pH in the recommended level for attaining optimum survival and growth of *O. niloticus*. These results suggest that, feeding *O.niloticus* with MI and RB diets in fertilized ponds does not significantly affect water quality parameters.

The present study has shown dominance of phytoplankton by Euglenophyceae, Bacillariophyceae, Chlorophyceae and Cyanophyceae in the water ponds. The present findings are corroborated by results reported by Degefu et al. (2011), Mette et al. (2011), Ssanyu et al. (2011) and Shahabuddin et al. (2012). The results further showed statistically comparable phytoplankton biomass and abundance between the ponds under MI and RB treatments. These findings are in agreement with the results reported by Abou et al. (2013) for *O. niloticus* reared in ponds and fed a plant-based diet (*Azolla* spp.). The comparability in composition, biomass and abundance of phytoplankton in the ponds is due to similar diets and fertilization rates used. During the present study, the ponds in the two sets were maintained with diets which had comparatively similar nutrient composition and were equally fertilized with similar amounts of cattle manure. Consequently, uneaten feeds and cattle manure used in the fish ponds stimulated and favoured growth of similar types of phytoplankton species at equal biomass and abundance.

The present results showed statistically comparable growth performance, survival and condition factors between *O.niloticus* fed on MI and RB diets. The final mean weights obtained in the present study for *O. niloticus* fed on MI (128.32 ± 7.32 g) and those fed on RB (115.06 ± 6.53 g) and daily weight gain of 0.43 ± 0.03 and 0.38 ± 0.03 g day^−1^ for *O. niloticus* fed on MI and RB respectively are comparable with final mean weight of 108.20 ± 15.40 g and daily weight gain of 0.29 g day^−1^ reported by Dagne et al. (2013) for mixed-sex *O.niloticus* fed on wheat bran. The percentage survivals of 82.60 ± 4.53 % and 81.54 ± 6.18 % and the condition factors of 1.85 ± 0.02 and 1.89 ± 0.02 for *O. niloticus* fed on MI and RB diets respectively are in agreement with survival rates of 83.24 ± 0.24 % and condition factor range of 1.69–1.70 obtained by Opiyo et al. (2014) for *O.niloticus* fed on a commercial diet.

The comparability in growth performance, survival and condition factor between *O.niloticus* fed on MI and RB diets is explained by the relatively similar nutrient composition of the two diets used. The nutrient composition of two diets used in the present study compares well save for slight differences in protein and lipid. The MI diet had relatively higher protein whereas RB diet had comparatively higher lipid. Dietary protein is an important nutrient in promoting fast growth of *O.niloticus* (Abdel-Tawwab et al. 2010). Similarly, Ahmad (2008) elucidated that, growth of fish is optimized by increasing the amount of other dietary energy sources such as lipids. Lipids contain more energy per unit weight than protein and carbohydrates (Jauncey 1998), are used efficiently as energy sources (De Silva and Anderson 1995) and are digested and metabolized easily by fish (Doreau and Chilliard 1997). Previous studies have established that, proper nutrient composition of feeds is a primary factor required for survival, muscle development and growth performance of *O. niloticus* (Abou et al. 2013; Valente et al. 2013; Ferdous et al. 2014; Wijaya et al. 2015). Accordingly, the slight differences in protein and lipid between the two diets used ensured similar survival, condition factor and growth of *O.niloticus* because they balanced their effect. The MI diet promoted survival, increased condition factor and growth of *O.niloticus* by its relatively higher protein content while the comparatively higher lipid content in RB diet was also effectively utilized by the fish to promote growth, survival and similar condition factor. These results imply that, farmers can use either of the diets without affecting survival, condition factor and growth performance of their fish in fertilized ponds.

Furthermore, phytoplankton biomass stimulated by decomposed uneaten diets and animal manure fertilizer used in the ponds also caused statistically comparable *O.niloticus* survival, condition factor and growth (Abdel-Tawwab et al. 2007). *O*. *niloticus* is known to feed on phytoplankton available in the water when cultured in semi-intensive earthen ponds (Abou et al. 2013). The high nutritional value of phytoplankton in ponds generally account for a large share of total fish survival, condition factor and growth. Phytoplankton contain significant amount of protein ranging from 120 to 350 g kg^−1^, lipids 72 to 230 g kg^−1^ and carbohydrates from 82 to 87 g kg^−1^ on a dry matter basis (Kang’ombe et al. 2006). Likewise, Abou et al. (2013) found the levels of protein, carbohydrate and lipid in natural food organisms to be as higher as 511, 273 and 77 g kg^−1^ respectively. According to Schroeder et al. (1990), natural foods including phytoplankton contribute for about 50–80 % in *O.niloticus* growth cultured in semi-intensive systems. The predominant phytoplankton species recorded in the present study (Bacillariophyceae Chlorophyceae, Cyanophyceae and Euglenophyceae) all form important diet for *O.niloticus* (Uddin et al. 2007; Abdel-Tawwab 2011). Consequently, the similar composition, biomass and abundance of phytoplankton in pond water of *O.niloticus* fed on the MI and RB diets led to comparable survival, condition factor and growth of *O. niloticus*.

In addition, comparable survival, condition factor and growth performance between the two treatments is attributed to similar water quality parameters. It has been established that, the overall survival, condition factor and growth performance of cultured fish partly depends on water quality (Shoko et al. 2014a). Water quality parameters such as dissolved oxygen, water temperature and pH affect growth of *O.niloticus* because they are required for optimum feeding, growing and excretion of wastes in water (Dampin et al. 2012; Dagne et al. 2013). The main water quality parameters measured in this study did not differ significantly between the two dietary treatments and were within the optimum range for *O.niloticus* rearing (Suresh 2003; El-Sayed 2006; Alabaster and Lloyd 2013). Consequently, they did not cause any significant variation in survival, condition factor and growth performance.

The present study showed that, the use of either MI or RB diet did not affect the yield performance of *O.niloticus*. The net (3459.61 ± 874.67 and 2976.10 ± 261.39 kg ha^−1^ year^−1^) and gross annual yields (4933.32 ± 1230.24 and 3371.47 ± 261.39 kg ha^−1^ year^−1^) for *O.niloticus* fed on MI and RB diets respectively are contrastingly higher than 1421 and 2437 kg ha^−1^ year^−1^ respectively reported by Dagne et al. (2013) for mixed-sex *O.niloticus*. These discrepancies could be attributed to differences in stocking density, feeding, pond fertilization and study duration. In Dagne et al. (2013) study *O.niloticus* were stocked at two fish m^−2^ and fed on wheat bran alone for a period of 240 days whereas in the present study the fish were stocked at three fish m^−2^ and fed on MI and RB diets with the addition of manure fertilization for a period of 270 days.

Yield is determined by growth rate of the cultured fish (Mridha et al. 2014), their survival rate (Al-Hafedh and Ali 2004) and the state of water quality parameters in the culture system (Ibrahim and Naggar 2010). Variation in these parameters significantly affect fish yield from an aquaculture system (Cao Quoc et al. 2012). The similarity in yields between *O.niloticus* fed on MI and RB diets is related to comparable growth, survival rate as well as condition factor and water quality parameters. In the present study, the above mentioned factors did not differ significantly between *O.niloticus* fed on the two dietary treatments. Thus, farmers who can afford either mixed ingredients (MI) or rice bran alone (RB) can use them without significantly affecting the yield of *O.niloticus* in fertilized ponds.

## Conclusion

The present study has revealed that the use of either mixed ingredients or rice bran alone diet does not significantly affect growth performance, survival, condition factor and yield of *O.niloticus* cultured in semi-intensive earthen ponds in which fertilization using cattle manure to stimulate natural food organisms is practised. The use of the two diets resulted in similar water quality parameters and phytoplankton composition, biomass and abundance which caused similarity in growth performance, survival, condition factor and yield of the cultured *O.niloticus*. Under semi-intensive culture conditions, *O.niloticus* farmers are advised to use rice bran which is readily available for free or at low cost in their localities and fertilize their ponds with cattle manure.
